# Association of circulating biomarkers with illness severity measures differentiates myalgic encephalomyelitis/chronic fatigue syndrome and post-COVID-19 condition: a prospective pilot cohort study

**DOI:** 10.1186/s12967-024-05148-0

**Published:** 2024-04-10

**Authors:** Joan Carles Domingo, Federica Battistini, Begoña Cordobilla, Maria Cleofé Zaragozá, Ramón Sanmartin-Sentañes, Jose Alegre-Martin, Trinitat Cambras, Jesus Castro-Marrero

**Affiliations:** 1 Department of Biochemistry and Molecular Biomedicine, Faculty of Biology, University of Barcelona, Barcelona, 08028 Spain; 2 Molecular Modelling and Bioinformatics Group, Institute for Research in Biomedicine, Barcelona Institute of Science and Technology, Barcelona, 08028 Spain; 3 Clinical Research Department, Laboratorios Viñas, Barcelona, 08012 Spain; 4 Division of Rheumatology, Clinical Unit in ME/CFS and Long COVID, Vall d’Hebron University Hospital, Universitat Autònoma de Barcelona, Barcelona, 08035 Spain; 5 Division of Rheumatology, Research Unit in ME/CFS and Long COVID, Vall d’Hebron Research Institute, Universitat Autònoma de Barcelona, Barcelona, 08035 Spain; 6 Department of Biochemistry and Physiology, Faculty of Pharmacy and Food Sciences, University of Barcelona, Barcelona, 08028 Spain

**Keywords:** Biomarkers, Chronic fatigue syndrome, Endothelial dysfunction, Inflammation, Long COVID, Myalgic encephalomyelitis, Post-acute sequelae of COVID-19, Post-exertional malaise

## Abstract

**Background:**

Accumulating evidence suggests that autonomic dysfunction and persistent systemic inflammation are common clinical features in myalgic encephalomyelitis/chronic fatigue syndrome (ME/CFS) and long COVID. However, there is limited knowledge regarding their potential association with circulating biomarkers and illness severity in these conditions.

**Methods:**

This single-site, prospective, cross-sectional, pilot cohort study aimed to distinguish between the two patient populations by using self-reported outcome measures and circulating biomarkers of endothelial function and systemic inflammation status. Thirty-one individuals with ME/CFS, 23 individuals with long COVID, and 31 matched sedentary healthy controls were included. All study participants underwent non-invasive cardiovascular hemodynamic challenge testing (10 min NASA lean test) for assessment of orthostatic intolerance. Regression analysis was used to examine associations between outcome measures and circulating biomarkers in the study participants. Classification across groups was based on principal component and discriminant analyses.

**Results:**

Four ME/CFS patients (13%), 1 with long COVID (4%), and 1 healthy control (3%) presented postural orthostatic tachycardia syndrome (POTS) using the 10-min NASA lean test. Compared with matched healthy controls, ME/CFS and long COVID subjects showed higher levels of ET-1 (*p* < 0.05) and VCAM-1 (*p* < 0.001), and lower levels of nitrites (NOx assessed as NO_2_^-^ + NO_3_^-^) (*p* < 0.01). ME/CFS patients also showed higher levels of serpin E1 (PAI-1) and E-selectin than did both long COVID and matched control subjects (*p* < 0.01 in all cases). Long COVID patients had lower TSP-1 levels than did ME/CFS patients and matched sedentary healthy controls (*p* < 0.001). As for inflammation biomarkers, both long COVID and ME/CFS subjects had higher levels of TNF-α than did matched healthy controls (*p* < 0.01 in both comparisons). Compared with controls, ME/CFS patients had higher levels of IL-1β (*p* < 0.001), IL-4 (*p* < 0.001), IL-6 (*p* < 0.01), IL-10 (*p* < 0.001), IP-10 (*p* < 0.05), and leptin (*p* < 0.001). Principal component analysis supported differentiation between groups based on self-reported outcome measures and biomarkers of endothelial function and inflammatory status in the study population.

**Conclusions:**

Our findings revealed that combining biomarkers of endothelial dysfunction and inflammation with outcome measures differentiate ME/CFS and Long COVID using robust discriminant analysis of principal components. Further research is needed to provide a more comprehensive characterization of these underlying pathomechanisms, which could be promising targets for therapeutic and preventive strategies in these conditions.

## Background

Myalgic encephalomyelitis, commonly referred to as chronic fatigue syndrome (ME/CFS), and post-acute sequelae of SARS-CoV-2 infection (PASC), also known as long COVID, are complex, multifaceted, and poorly understood disabling conditions. Research is currently ongoing to uncover their underlying pathomechanisms [1, 2]. Both ME/CFS and long COVID present challenges with regard to diagnosis, as in neither case are there clear diagnostic criteria or specific biomarkers and nor is there currently a cure and treatment for these conditions. Although the precise etiology underlying their lingering symptoms remains largely unknown, several hypotheses have been formulated regarding their causes, none of which are mutually exclusive to ME/CFS [3–5] or long COVID [1, 6].

Following acute SARS-CoV-2 infection, a subset of individuals continues to suffer from lingering symptoms very similar to those associated with ME/CFS. It has been estimated that about half of people presenting with long COVID at six months meet the case criteria for ME/CFS [7, 8]. Importantly, the large increase in ME/CFS prevalence due to COVID-19 is expected by 2050 to leave 150 million people affected worldwide, posing a considerable challenge for global healthcare systems [9]. Early results from mechanistic studies in long COVID suggest a constellation of cardinal symptoms and underlying biological abnormalities very similar to those found in ME/CFS such as new onset post-exertional malaise that is not substantially alleviated by rest and which lasts from days to even weeks to months, as well as unrefreshing sleep, cognitive impairment, autonomic dysfunction, and gastrointestinal complaints that persist for more than six months, based on the updated case criteria for both conditions [8].

There is emerging evidence that lingering endothelial dysfunction and low-grade systemic inflammation are common clinical features of both conditions [10–13], although the specific pathophysiological mechanisms and their interactions have yet to be fully elucidated. Furthermore, studies in ME/CFS and long COVID display significant heterogeneity and inconsistent findings with regard to the association between putative biomarkers and illness severity status [14, 15]. Accurate biomarkers for the diagnosis of ME/CFS and long COVID are therefore urgently needed [11, 16–18]. Longitudinal studies involving large cohorts can broaden our understanding of the natural course of these conditions, and may also help to identify potential biomarkers in patient subgroups. One of the many questions that remain unanswered is whether ME/CFS and long COVID share similar phenotypes underlying their common pathophysiological mechanisms [2, 8].

The purpose of this study is to (1) describe baseline demographic and clinical characteristics in a Spanish cohort of individuals with ME/CFS and long COVID, (2) assess orthostatic intolerance in study participants using the 10-min NASA lean test, (3) identify a panel of circulating biomarkers related to endothelial dysfunction and inflammation among participants, (4) differentiate between ME/CFS and long COVID based on the circulating biomarker profile and self-reported outcome measures, and (5) examine possible associations between the assessed biomarkers and illness severity to help further development of new pharmacological targets for these conditions.

## Methods

### Study population

A unicenter, prospective, cross-sectional, pilot cohort study was conducted involving 31 ME/CFS patients (mean age: 49.3 ± 3.1 years; 65% female), 23 individuals with long COVID (mean age: 48.7 ± 2.4 years; 65% female), and 31 matched healthy sedentary controls (mean age: 41.7 ± 1.8 years; 71% female) recruited consecutively between September 2020 and December 2022 from the largest outpatient tertiary referral center in Spain (ME/CFS Clinical Unit, Vall d’Hebron University Hospital, Barcelona, Spain). After receiving verbal and written information on the study protocol, they all signed informed consent to participate prior to enrollment. The study protocol was approved by the Institutional Review Board of Vall d’Hebron University Hospital (reference number PR/AG 201/2016), and all procedures were conducted in accordance with the ethical standards of the board and the 1964 Declaration of Helsinki including its later amendments.

### Eligibility criteria

ME/CFS patients were eligible if they were aged ≥ 18 years and had a confirmed diagnosis by a specialist physician based on international consensus criteria (2011 ICC), the recommended case criteria for ME/CFS research purposes being those set out in an updated EUROMENE report [19].

COVID-19 “long haulers” (a.k.a. long COVID) were eligible for enrollment if they were aged ≥ 18 years and, following a confirmed diagnosis of acute COVID-19 infection based on a positive SARS-CoV-2 RT-PCR test on nasal swab during the COVID-19 pandemic, were still suffering from persistent, unexplained symptoms/signs ≥ 3 months after the acute COVID-19 infection, as established in the 2021 WHO clinical case definition for post-COVID-19 condition [6].

Healthy sedentary volunteers were eligible if they had neither experienced any self-reported autonomic symptoms nor had been in recent contact with anyone who was infected with SARS-CoV-2 (COVID-19) within 90 days prior to the study, no significant neurological, cardiac, endocrine or neuroimmune disorders, no alcohol or drug dependence, and did not use daily prescribed medications. All healthy sedentary subjects were recruited through word-of-mouth from the local community and did not meet the case criteria for either ME/CFS or long COVID at the time of enrollment.

All participants with cardiovascular dysautonomia were diagnosed by a physician based on consensus criteria from the POTS Working Group for the U.S. NIH [20]. All participants were of Caucasian descent, from the same geographical area, and had a sedentary lifestyle at the time of the study. They were subject to stringent exclusion criteria, as previously described by our group [21, 22]. The major exclusion criteria were a relevant previous or current diagnosis of an autoimmune disorder, multiple sclerosis, psychosis, major depression disorder, heart disease, hematological disorders, infectious diseases, sleep apnea or metabolic disorders; pregnancy or breast-feeding; smoking habit; strong hormone-related drugs; and preexisting fatigue-associated symptoms or evidence of multi-organ failure that did not meet the case criteria for ME/CFS and long COVID used in this study. Baseline demographic and clinical characteristics of the study population are displayed in Table 1.

**Table 1:** Baseline demographic and clinical characteristics of the study participants

Variables	ME/CFS	Long COVID	Healthy controls	*P*-value ^1^	*P*-value ^2^
	(*n* = 31)	(*n* = 23)	(*n* = 31)		
Age, years	49.3 ± 3.1	48.7 ± 2.4	41.7 ± 1.8	n.s.	n.s.
Female, n (%)	20 (65)	15 (65)	22 (71)	n.s.	n.s.
BMI, kg/m^2^	25.5 ± 0.6	25.8 ± 0.9	24.2 ± 0.6	n.s.	n.s.
Illness duration at the inclusion time, years	7.4 ± 0.7	2.1 ± 0.6	n/a	n/a	n/a
SBP, mmHg	128.1 ± 3.1	124.6 ± 2.9	115.6 ± 2.0	**< 0.0001**	**0.0038**
DBP, mmHg	81.8 ± 1.5	77.8 ± 1.3	71.4 ± 1.4	**< 0.0001**	**0.0006**
HR, bpm	73.6 ± 1.8	67.2 ± 2.2	65.5 ± 1.7	**0.0036**	n.s.
**Self-reported symptoms, n (%)**					
Post-exertional malaise	25 (80)	23 (100)	0 (0)	**< 0.0001**	**< 0.0001**
Cognitive impairments	6 (19)	23 (100)	0 (0)	**0.024**	**< 0.0001**
Unrefreshing sleep	22 (70)	20 (87)	0 (0)	**< 0.0001**	**< 0.0001**
Headache	5 (16)	17 (74)	0 (0)	**0.050**	**< 0.0001**
Anxiety/depression	10 (32)	6 (26)	0 (0)	**0.0008**	**0.004**
Gastrointestinal disturbances	10 (32)	14 (61)	0 (0)	**0.0008**	**< 0.0001**
Orthostatic intolerance	10 (32)	6 (26)	0 (0)	**0.0008**	**0.004**
**Pre-existing comorbidities, n (%)**					
Fibromyalgia	13 (41)	7 (30)	0 (0)	**< 0.0001**	**0.0014**
Hypertension	7 (22)	5 (22)	0 (0)	**0.011**	**0.011**
IBS	4 (12)	4 (17)	0 (0)	**0.017**	**0.028**
Hypothyroidism	4 (12)	0 (0)	0 (0)	**< 0.0001**	n.s.
Dyslipidemia	4 (12)	4 (17)	0 (0)	n.s.	**0.028**
Diabetes	4 (12)	3 (13)	1 (3)	n.s.	n.s.
Cardiovascular disease	0 (0)	5 (22)	0 (0)	n.s.	**0.011**
Cerebrovascular disorder	0 (0)	0 (0)	0 (0)	n.s.	n.s.
Chronic COPD/asthma	4 (12)	1 (4)	0 (0)	n.s.	n.s.
CKD	0 (0)	1 (4)	0 (0)	n.s.	n.s.
**COVID-19 status, n (%)**					
At home	14 (45)	4 (17)	5 (16)	**0.026**	n.s.
Outpatients	1 (3)	14 (61)	5 (16)	n.s.	**0.0012**
Hospitalized	0 (0)	5 (22)	0 (0)	n.s.	**0.011**
**COVID-19 wave, n (%)** ^**a**^					
Wave 1	20 (65)	19 (83)	0 (0)	**< 0.0001**	**< 0.0001**
Wave 2	6 (19)	4 (17)	5 (16)	n.s.	n.s.
Wave 3	3 (9)	0 (0)	7 (23)	n.s.	**0.016**
**Vaccination status, n (%)** ^**b**^					
None dose	1 (3)	1 (4)	0 (0)	n.s.	n.s.
One dose	30 (96)	22 (96)	31 (100)	n.s.	n.s.
Two doses	30 (96)	16 (70)	30 (96)	n.s.	**0.0078**
Three or more doses	19 (61)	8 (35)	22 (70)	n.s.	**0.013**
**Vaccine type, n (%)**					
Pfizer	20 (64)	17(74)	24 (77)	n.s.	n.s.
Oxford	5 (16)	4 (17)	5 (16)	n.s.	n.s.
Moderna	4 (12)	1 (4)	2 (7)	n.s.	n.s.
Janssen	1 (3)	0 (0)	0 (0)	n.s.	n.s.
**COVID-19 severity** ^**c**^					
Asymptomatic	20 (64)	5 (22)	29 (94)	**0.011**	**< 0.0001**
Mild/moderate	10 (32)	18 (78)	2 (6)	**0.022**	**< 0.0001**
Severe	1(4)	0 (0)	0 (0)	n.s.	n.s.
Critically severe	0 (0)	0 (0)	0 (0)	n.s.	n.s.
**Measures** ^**d**^					
**FIS-40**					
Global score (0-160)	130.3 ± 3.3	127.5 ± 3.6	17.2 ± 2.6	**< 0.0001**	**< 0.0001**
Physical	34.2 ± 0.8	33.3 ± 1.0	5.1 ± 0.7	**< 0.0001**	**< 0.0001**
Cognitive	61.3 ± 2.0	59.1 ± 2.2	8.0 ± 1.3	**< 0.0001**	**< 0.0001**
Psychosocial	34.8 ± 0.9	35.2 ± 1.0	4.1 ± 0.7	**< 0.0001**	**< 0.0001**
**PSQI**					
Global score (0–21)	14.8 ± 0.7	14.0 ± 0.9	5.6 ± 0.4	**< 0.0001**	**< 0.0001**
Subjective sleep quality	2.2 ± 0.1	2.2 ± 0.1	0.7 ± 0.1	**< 0.0001**	**< 0.0001**
Sleep latency	2.1 ± 0.2	2.2 ± 0.2	1.3 ± 0.1	**< 0.0001**	**0.0003**
Sleep duration	1.9 ± 0.1	1.8 ± 0.2	1.2 ± 0.1	**< 0.0001**	**0.0003**
Habitual sleep efficiency	2.1 ± 0.2	1.8 ± 0.3	0.5 ± 0.1	**< 0.0001**	**< 0.0001**
Sleep disturbances	2.3 ± 0.1	2.0 ± 0.1	1.0 ± 0.04	**< 0.0001**	**< 0.0001**
Use of sleeping medication	2.0 ± 0.2	1.8 ± 0.3	0.3 ± 0.09	**< 0.0001**	**< 0.0001**
Daytime dysfunction	2.24 ± 0.1	2.1 ± 0.2	0.6 ± 0.08	**< 0.0001**	**< 0.0001**
**HADS**					
Global score (0–42)	24.8 ± 1.2	22.5 ± 1.7	6.5 ± 0.6	**< 0.0001**	**< 0.0001**
Anxiety	12.9 ± 0.6	11.2 ± 1.0	4.7 ± 0.4	**< 0.0001**	**< 0.0001**
Depression	11.9 ± 0.7	11.3 ± 0.9	1.8 ± 0.3	**< 0.0001**	**< 0.0001**
**COMPASS-31**					
Global score (0-100)	56.0 ± 2.2	40.9 ± 3.7	11.8 ± 1.3	**< 0.0001**	**< 0.0001**
Orthostatic intolerance	23.7 ± 1.2	17.2 ± 2.2	5.1 ± 0.8	**< 0.0001**	**< 0.0001**
Vasomotor	1.7 ± 0.2	1.3 ± 0.3	0.08 ± 0.1	**< 0.0001**	**< 0.0001**
Secretomotor	10.0 ± 0.5	7.5 ± 0.9	1.1 ± 0.3	**< 0.0001**	**< 0.0001**
Gastrointestinal	12.4 ± 0.7	8.6 ± 1.0	4.0 ± 0.5	**< 0.0001**	**< 0.0001**
Bladder	4.4 ± 0.5	3.5 ± 0.8	0.5 ± 0.1	**< 0.0001**	**< 0.0001**
Pupillomotor	3.8 ± 0.2	2.6 ± 0.2	1.0 ± 0.1	**< 0.0001**	**< 0.0001**
**OGS**					
Global score (0–20)	12.8 ± 0.9	10.6 ± 1.0	0.9 ± 0.3	**< 0.0001**	**< 0.0001**
Frequency of orthostatic symptoms	2.7 ± 0.2	2.2 ± 0.2	0.3 ± 0.1	**< 0.0001**	**< 0.0001**
Severity of orthostatic symptoms	2.6 ± 0.2	2.3 ± 0.2	0.3 ± 0.07	**< 0.0001**	**< 0.0001**
Conditions under which orthostatic symptoms occur	2.7 ± 0.2	2.0 ± 0.2	0.2 ± 0.07	**< 0.0001**	**< 0.0001**
Activities of daily living	2.5 ± 0.2	2.3 ± 0.3	0.02 ± 0.02	**< 0.0001**	**< 0.0001**
Standing time	2.3 ± 0.3	1.7 ± 0.2	0.12 ± 0.08	**< 0.0001**	**< 0.0001**
**SF-36**					
Global score (0-100)	27.0 ± 2.2	29.1 ± 3.0	86.9 ± 1.5	**< 0.0001**	**< 0.0001**
Physical functioning	34.3 ± 3.2	44.8 ± 5.6	97.1 ± 0.7	**< 0.0001**	**< 0.0001**
Physical role functioning	2.4 ± 1.5	1.1 ± 1.1	92.1 ± 2.5	**< 0.0001**	**< 0.0001**
Bodily pain	18.3 ± 2.4	29.6 ± 4.5	86.2 ± 2.4	**< 0.0001**	**< 0.0001**
General health perception	21.7 ± 2.4	27.8 ± 2.9	86.7 ± 1.5	**< 0.0001**	**< 0.0001**
Vitality	14.0 ± 2.7	20.0 ± 3.9	71.7 ± 2.3	**< 0.0001**	**< 0.0001**
Social role functioning	32.6 ± 3.6	33.4 ± 5.0	94.0 ± 1.8	**< 0.0001**	**< 0.0001**
Emotional role functioning	46.3 ± 6.9	34.8 ± 9.7	90.6 ± 3.2	**< 0.0001**	**< 0.0001**
Mental health	18.2 ± 2.5	44.6 ± 6.8	95.2 ± 3.4	**< 0.0001**	**< 0.0001**
Physical health component summary scores (PCS)	36.1 ± 3.7	21.2 ± 4.6	76.4 ± 2.2	**< 0.0001**	**< 0.0001**
Mental health component summary scores (MCS)	7.8 ± 3.1	11.2 ± 2.8	69.3 ± 2.6	**< 0.0001**	**< 0.0001**

Data are presented as mean ± standard error of the mean (SEM) or number of participants (percentages), unless otherwise indicated. *P*-values were calculated by Mann-Whitney *U*-tests for continuous variables and from Fisher’s exact test for categorical variables. Bold values denote statistical significance at *P* ≤ 0.05 between cohorts. Superscripts (^1^) and (^2^) are the *P*-values for ME/CFS vs. healthy controls and Long COVID vs. healthy controls, respectively. ^a^ First wave of the COVID-19 epidemic in Spain lasted from February 2020 to September 2020 (original wild-type variant), second wave runs from October 2020 to July 2021 (alpha variant), and three wave is from August 2021 to July 2022 (delta/omicron variants). ^b^ The average periods of the vaccine administration were January 2021 (first dose), February 2021 (second dose), and November 2021 (third dose). ^c^ The SARS-CoV-2 infection (COVID-19) severity was defined based on the 2021 NIH/CDC COVID-19 treatment guidelines (available at https://www.covid19treatmentguidelines.nih.gov/; accessed on 15 June 2023). ^d^ Baseline self-reported outcome measures of symptoms (global and domain scores), as explained in the *Methods* section. *n/a* not applicable, *n.s*. not significant

### Experimental procedures

All participants attended the aforementioned hospital between 8:00 a.m. and 11:00 a.m. for a face-to-face clinical assessment by a specialist physician. Baseline demographic and clinical characteristics of each participant were recorded based on self-reported outcome measures. A blood sample was taken from all participants for routine biochemical analysis and circulating biomarkers to assess endothelial dysfunction and systemic inflammation was assayed. All participants underwent the same cardiovascular orthostatic challenge protocol (10-min NASA lean test) to evaluate orthostatic intolerance.

### Assessment of cardiovascular autonomic function

Orthostatic intolerance was evaluated using a standardized passive standing test (10-min NASA lean test, NLT), a simple and well-established non-invasive orthostatic challenge protocol used to assess cardiovascular compensatory autonomic responses to standing. The NLT enables detection of orthostatic intolerance (OI) phenotypes such as orthostatic hypotension (OH) and postural orthostatic tachycardia syndrome (POTS) by measuring cardiovascular hemodynamic parameters (SBP/DBP and HR), and it is suitable for both clinical and research purposes [23].

Briefly, the NLT was conducted in a consistent manner by the same examiner in the morning between 8:30 a.m. and 11 a.m., in a quiet room with an average relative temperature of 22.3 ± 1.5 °C and humidity of 55.7 ± 4.8%. Participants were first asked to lie down on an exam table for 5 min and then to stand and lean against a wall, with their heels 15–20 cm away from the wall. An automated BP cuff with a monitor (Beurer BM-26, Beurer GmbH & Co., Ulm, Germany) was placed on the left arm, recording the systolic BP (SBP), diastolic (DBP), and heart rate (HR) at 1-min intervals. Baseline SBP/DBP HR were consecutively recorded twice during the supine position and every minute during the full 10 min after attaining the upright position. Throughout the recording, participants stood with only their shoulder blades touching the wall and their heels were positioned 15–20 cm from the wall. Participants were asked to remain still, and any talking or movement was discouraged, except for reporting any symptoms of concern. The NLT was stopped early at the request of the subject, or in the event of severe pre-syncope. After 10 min upright, each participant was asked about the frequency/severity and impact of orthostatic symptoms on a 5-item OGS score [23].

### Definitions of hemodynamic parameters recorded during the orthostatic challenge test

Criteria for OI were based on the 2021 expert consensus statement and guidelines for the definition of OH and POTS, as follows: (1) OH was defined as a decrease in SBP of ≥ 20 mmHg, or a decrease in DBP of ≥ 10 mmHg in the first 3 min standing, compared with resting supine values; and (2) POTS was defined as an increase in HR ≥ 30 bpm and/or a current HR ≥ 120 bpm without BP changes, based on the average of the final 3 min standing. Orthostatic intolerance (OH and POTS) during the 10-min NLT was quantified as the difference between supine and standing hemodynamic changes (ΔBP and ΔHR), as previously described [20, 24, 25]. For the purposes of this study, SBP/DBP and HR were used as maximum raw values recorded during the orthostatic test. For correlation analysis and group comparison, we calculated mean values during the final 2 min in the supine position (sp), mean values during the first 3 min standing (3 F), and mean values of final 3 min (3 L). Changes in these variables compared with values in the supine position (baseline) were also calculated (Δ3F or Δ3L).

### Measures

Participants were asked to fill out a set of validated self-administered screening questionnaires regarding their current health status one week after the first clinical assessment. Changes in perceived fatigue (Fatigue Impact Scale, FIS-40), sleep quality (Pittsburgh Sleep Quality Index, PSQI), anxiety and depression symptoms (Hospital Anxiety and Depression Scale, HADS), autonomic dysfunction symptoms (Composite Autonomic Symptom Score-31, COMPASS-31), frequency and severity of orthostatic symptoms (Orthostatic Grading Scale, OGS), and health-related quality of life (Short-Form Health Survey, SF-36) were rated by each study participant under the supervision of two trained investigators (J.A.-M. and J.C.-M.), who oversaw compliance as described in our previous study [22].

### Perceived fatigue

Fatigue severity was assessed using the 40-item FIS-40. This questionnaire assesses three domains reflecting the perceived feeling of fatigue: physical (10 items), cognitive (10 items), and psychosocial functions (20 items). Each item is rated from 0 (no fatigue) to 4 (severe fatigue), and a total score is calculated by summing the item scores (range 0 to 160). Higher scores indicate greater functional limitations in daily life due to fatigue [26].

### Sleep disturbances

Sleep quality disturbances were evaluated with the 19-item PSQI, which assesses seven domains: subjective sleep quality, sleep latency, sleep duration, habitual sleep efficiency, sleep perturbations, use of sleeping medication, and daytime dysfunction. The overall PSQI score ranges from 0 to 21 points, with scores ≥ 5 indicating poorer sleep quality [27].

### Anxiety and depression

The HADS is a self-report scale used to screen for the presence of anxiety/depression symptoms in people with chronic illnesses. It consists of a 14-item inventory scored on a four-point Likert-type scale (from 0 to 3) and divided into two subscales, anxiety (7 items) and depression (7 items), which are scored independently. Each subscale score ranges from 0 to 21, and the higher the score, the greater the level of anxiety or depression [28].

### Autonomic dysfunction

To assess dysautonomia, all participants were screened using the COMPASS-31, a questionnaire designed to evaluate the frequency and severity of autonomic function symptoms in six core domains: orthostatic intolerance (four items), vasomotor (three items), secretomotor (four items), gastrointestinal (twelve items), bladder (three items), and pupillomotor symptoms (five items). The six domain scores are summed to provide a global COMPASS-31 score ranging from 0 (no symptoms) to 100 (worst symptoms). Higher scores indicate more severe autonomic complaints [29].

### Orthostatic grading scale

The orthostatic grading scale (OGS) is a validated 5-item self-report questionnaire designed to assess symptoms of orthostatic intolerance due to cardiovascular orthostatic dysfunction. The five questions address the frequency/severity of and interference due to orthostatic symptoms in daily life activities. Respondents rate each item on a scale of 0 to 4. Item scores are summed to give an overall OGS score ranging from 0 (no orthostatic symptoms) to 20 (worst orthostatic symptoms). Higher scores indicate greater severity of orthostatic intolerance [30].

### Short-form health survey (SF-36)

The SF-36 is a generic scale that provides a health status profile, and it was used here to assess quality of life in study participants. Its 36 items explore eight dimensions of health status (physical function, role limitations due to physical health, bodily pain, general health, vitality, social functioning, emotional role, and mental health), and it yields summary scores for the physical and mental components [31].

### Blood collection and processing

After a 12-hour overnight fasting, 20 ml of peripheral whole blood were collected from each participant by venipuncture (from an antecubital vein with a 19-gauge needle) into SST-tubes and K_2_EDTA-containing tubes (BD Vacutainer, Becton Dickenson, Sarstedt, Barcelona, Spain). This was performed between 8:00 a.m. and 10:00 a.m. by a trained phlebotomy nurse (USIC Outpatient Clinical Unit, Vall d’Hebron University Hospital, Barcelona, Spain).

One tube was transported and delivered to the local core laboratory within 2 h of collection for routine blood tests/analyses, following standard and recommended procedures. All other blood samples were immediately centrifuged at 2,500 rpm for 15 min at 4 °C followed by a second centrifugation step under the same conditions (Thermo Scientific, Waltham, MA, USA), after which serum and plasma specimens were collected and stored in aliquots at − 80 °C until further assays. No sample was thawed more than twice. Repeated samples from each participant were measured in the same analytical batch.

### Measurement of endothelial function biomarkers

Blood levels of vascular endothelium proteins, namely soluble endothelin-1 (ET-1), thrombospondin-1 (TSP-1), vascular cell adhesion molecule-1 (VCAM-1), intracellular adhesion molecule-1 (ICAM-1), plasminogen activator inhibitor-1 (serpin E1/PAI-1), endothelial selectin, and adiponectin, as well as nitric oxide (NO_X_ assessed as NO_2_^-^ + NO_3_^-^), were measured to assess peripheral vascular endothelium function. Plasma concentrations of each protein were assayed in each participant with commercially available ELISA kits, according to the manufacturer’s instructions (Quantikine R&D Systems, Minneapolis, USA), using a Synergy™ H1M, hybrid multi-mode microplate reader (BioTek Instruments, Inc., Winooski, USA) at O.D. 450 nm. Results were analyzed by comparison with standard calibration curves in each well, and are presented as averages of two duplicated plasma samples.

### Measurement of inflammatory cytokines/chemokines levels

Twelve cytokines/chemokines, namely IL-1β, IL-4, IL-6, IL-8, IL-10, IL-13, TNF-α, monocyte chemoattractant protein-1 (MCP-1/CCL2), interferon-gamma (IFN-γ), interferon gamma-induced protein-10 (IP-10/CXCL10), macrophage inflammatory protein-1-alpha (MIP-1α), and leptin were measured simultaneously in duplicate plasma samples using a fluorescently labeled microsphere-based multiplex bead-array immunoassay, according to the manufacturer’s instructions (Cat# HCCBP1MAG-58 K and Cat# HCYTA-60 K, Milliplex Map Human Cytokines/Chemokines Magnetic Bead Panel I/II, Linco Research Millipore, Billerica, MA, USA). Measurements were taken on a Luminex-100 ISv2 reader (Linco Research Millipore, Billerica, MA, USA). For each cytokine/chemokine the standard curve ran from 3.2 to 10,000 pg/mL. Results were recorded in pg/mL based on standard curve values. The intra- and inter-assay coefficients of variation for each cytokine/chemokine were, respectively: IL-1β: 7% and 12%; IL-4: 3% and 11%; IL-6: 9% and 5%; IL-8: 7% and 5%; IL-10: 2% and 11%; IL-13: 2% and 11%; TNF-α: 8.7% and 4.9%; MCP-1: 2% and 11%; IFN-γ: 8% and 13%; IP-10: 7% and 11%; MIP-1α: 7% and 13%; and leptin: 5% and 7%.

### Statistical analysis and data integration

Data for continuous variables are reported as mean ± standard error of the mean (SEM), while for categorical variables we report numbers and percentages. Due to a limited sample size and skewed distribution, statistical comparisons were performed using non-parametric methods. Specifically, Fisher’s exact test was used for categorical variables, while for continuous variables we used either the Kruskal-Wallis test with Dunn´s post-hoc multiple comparison test or the Mann-Whitney U (rank sum) test for two groups. The Kolmogorov-Smirnov and Shapiro-Wilk tests were used to assess normality in the data distributions of the studied parameters. Box plots show mean ± SEM, including 25th and 75th percentiles of triplicate sampling. Statistical analyses were performed using GraphPad Prism software (GraphPad Prism 10.1.0.316 for Windows, serial number: GPS-1,359,963-L###-###; machine ID: E32AAD88B97; Boston, MA, USA). A two-tailed *p*-value ≤ 0.05 was considered statistically significant.

For the principal component analysis (PCA) we used the prcomp and autoplot function from the ggfortify library in R [32]. Autoplotting was used for both cluster analysis and representation of the different ellipses with coverage probability for a 95% confidence interval. The overlap between ellipses was calculated using the shipunov package in R, again with a 95% confidence interval. For the correlation heatmap we used the R package for multi-environment trial analysis (metan), selecting the Spearman’s method and a cut-off *p*-value < 0.05 [33].

Finally, in order to differentiate between the groups, we conducted a MANOVA followed by discriminant analysis. The discriminant function analysis evaluates canonical discriminant functions based on combinations of the selected markers which contribute maximally to group separation, and it assesses how well these functions discriminate the diagnosis. Examining Wilk’s lambda values for each of the predictors reveals how important the independent variable is to the discriminant function, with smaller values representing greater importance. Data were analyzed using IBM SPSS Statistics for Windows 27.0 (IBM Corp., Armonk, NY, USA).

## Results

### Clinical characteristics of study population

Table 1 summarizes the baseline demographic and clinical characteristics of study participants recruited in this study. Patients (two groups) and controls did not differ in terms of age, gender or BMI. The mean duration of illness was 7 years for ME/CFS and 2 years for long COVID patients. As shown in Table 1, all patients with ME/CFS and long COVID presented with a wide range of persistent symptoms, with no hospitalizations during the acute COVID-19 infection. Thirty of the 31 ME/CFS individuals and 22 of the 23 long COVID subjects (96.8% and 95.7%, respectively), as well as all matched sedentary healthy controls, were vaccinated with at least one dose against COVID-19 using Comirnaty (Pfizer-BioNTech) ≤ 8 months prior to study inclusion. The ME/CFS and long COVID groups differed from healthy subjects in their scores on self-reported outcome measures, with patients reporting more fatigue, more anxiety/depression symptoms, poorer sleep quality, greater dysautonomia, and lower health-related quality of life (all *p* < 0.001).

### Cardiovascular hemodynamic factors using the orthostatic challenge test

**Fig. 1:**
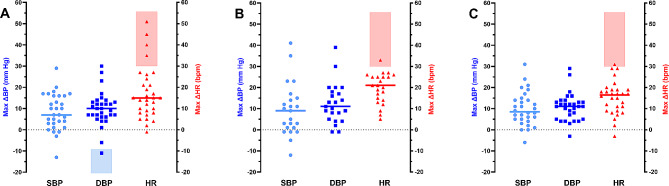
Cardiovascular hemodynamic response determinants to autonomic challenge test in the study population. Maximum increase in blood pressure (max ΔBP) and maximum increase in heart rate (max ΔHR) measured during the 10-min NASA lean test from supine position in the study participants. Panel (**A**) represents the maximum values for SBP/DBP and HR in ME/CFS patients, (**B**) maximum values for long COVID patients, and (**C**) maximum values for healthy sedentary controls. Each dot denotes a single participant and the horizontal lines represent the mean values for each hemodynamic parameter. Shaded bars indicate postural orthostatic hypotension (OH) and postural orthostatic tachycardia syndrome (POTS) in each study cohort, as defined in the *Methods* section

Table 2 summarizes data for the cardiovascular autonomic response variables assessed through the 10-min NLT. It can be seen that SBP and DBP in the supine position were significantly higher in the ME/CFS and long COVID groups than in matched sedentary healthy controls (all *p* < 0.05). Regarding HR, this was higher in ME/CFS patients than in matched sedentary healthy controls (*p* < 0.005), but no differences were observed between long COVID and healthy individuals on this variable. No differences by group were found for changes in cardiovascular hemodynamic variables. None of the participants had abnormal pulse pressure (less than 25% SBP), and this variable did not differ between groups. Assessment of orthostatic intolerance using the 10-min NLT indicated that 4 ME/CFS patients (13%), 1 with long COVID (4%), and 1 healthy control (3%) presented POTS. No differences between the groups in terms of distribution were found (Fig. 1A-C).

**Table 2:** Cardiovascular autonomic response variables recorded during the orthostatic stand test in the study population

Variables	ME/CFS (*n* = 31)	Long COVID (*n* = 23)	HCs (*n* = 31)	*P*-values
SBP_sp, mmHg	128.1 ± 3.1	124.6 ± 2.9	115.6 ± 2.0	**< 0.0001** ^**1**^ **/0.0038** ^**2**^
ΔSBP_3F, mmHg	0.2 ± 1.4	0.72 ± 2.25	3.1 ± 1.1	n.s./n.s.
DBP_sp, mmHg	81.8 ± 1.5	77.8 ± 1.3	71.4 ± 1.4	**< 0.0001** ^**1**^ **/0.0006** ^**2**^
ΔDBP_3F, mmHg	6.5 ± 1.5	6.57 ± 1.22	7.0 ± 1.0	n.s./n.s.
HR_sp, bpm	73.6 ± 1.8	67.2 ± 2.2	65.5 ± 1.7	**0.0036**^**1**^/n.s.
ΔHR_3L, bpm	12.7 ± 2.0	17.08 ± 1.92	13.9 ± 1.9	n.s./n.s.
PP_sp, mmHg	46.8 ± 2.0	46.8 ± 2.08	41.8 ± 1.4	n.s./n.s.
NPP_sp, %	36.1 ± 0.9	37.2 ± 0.87	36.1 ± 0.9	n.s./n.s.

Data for each hemodynamic parameter are displayed as mean ± SEM. *P*-values were attained from ANOVA. Superscripts (^1^) and (^2^) show statistically significant *P*-values for comparison between ME/CFS vs. healthy controls and long COVID vs. healthy controls, respectively. Significant comparisons are highlighted in bold (n.s. denote not significant). *ME/CFS* myalgic encephalomyelitis/chronic fatigue syndrome, *HCs* healthy controls, *SBP_sp* systolic blood pressure in supine position, *ΔSBP_3F* variation of SBP in the first three minutes of standing, *DBP_sp* diastolic blood pressure in supine position, *ΔDBP_3F* variation of DBP in the first three minutes of standing, *HR_sp* heart rate in supine position, *ΔHR_3L* variation of HR in the last three minutes of standing, PP_sp pulse pressure in supine position, NPP_sp narrowed pulse pressure. These latter variables were calculated according to the consensus equation as follows PP = SBP-DBP and NPP = PP/SBP. A pulse pressure (PP) that is less than 25% of the SBP is inappropriately low or narrowed, whereas a PP of greater than 100% is high or widened

### Assessment of circulating biomarkers in ME/CFS and long COVID

#### Endothelial function markers

**Fig. 2:**
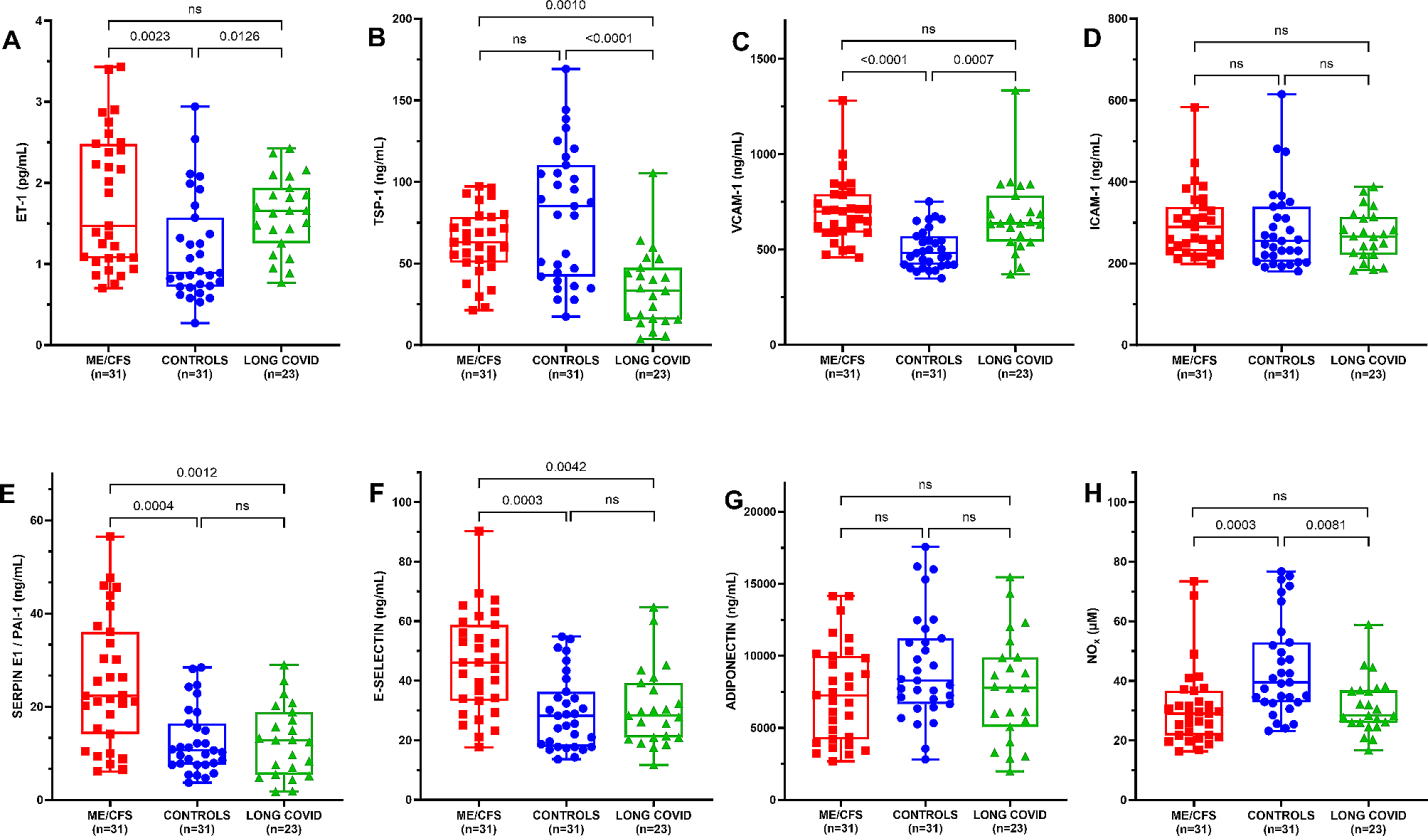
Circulating biomarkers of endothelial function in individuals with ME/CFS and long COVID compared with matched sedentary healthy controls. Representative box plot analysis of eight endothelial proteins showing statistically significant differences in plasma levels of ET-1, TSP-1, VCAM-1, ICAM-1, serpin E1 (PAI-1), E-selectin, adiponectin, and nitric oxide (NOx) at baseline from ME/CFS patients, individuals with long COVID, and matched sedentary healthy controls, as described in the *Methods* section (**panel A-H**). Each dot represents a single participant in each group. Values are expressed as mean ± standard error of the mean (SEM) of duplicated assays and are representative of two independent experiments. The box extends from the 25th to 75th percentiles, the line represents the mean, and the whiskers indicate the range of minimum and maximum values within SEM. *P*-values were calculated using the non-parametric Kruskal-Wallis test with Dunn’s post-hoc multiple comparison test. Significance level was set at *p < 0.05, **p < 0.01, and ***p < 0.001

As can be seen in Fig. 2, levels of endothelial biomarkers differed among the groups. Specifically, in comparison with matched sedentary healthy controls, patients with long COVID and those with ME/CFS both had higher concentrations of ET-1 and VCAM-1 (all *p* < 0.05) and lower levels of nitrites (NOx; *p* < 0.01). In addition, ME/CFS patients had higher levels of serpin E1 (PAI-1) and E-selectin than did individuals with long COVID and matched sedentary healthy controls (all *p* < 0.005). Finally, long COVID patients had lower TSP-1 concentrations than did ME/CFS patients and matched sedentary healthy controls (*p* < 0.001).

#### Inflammation status biomarkers

**Fig. 3:**
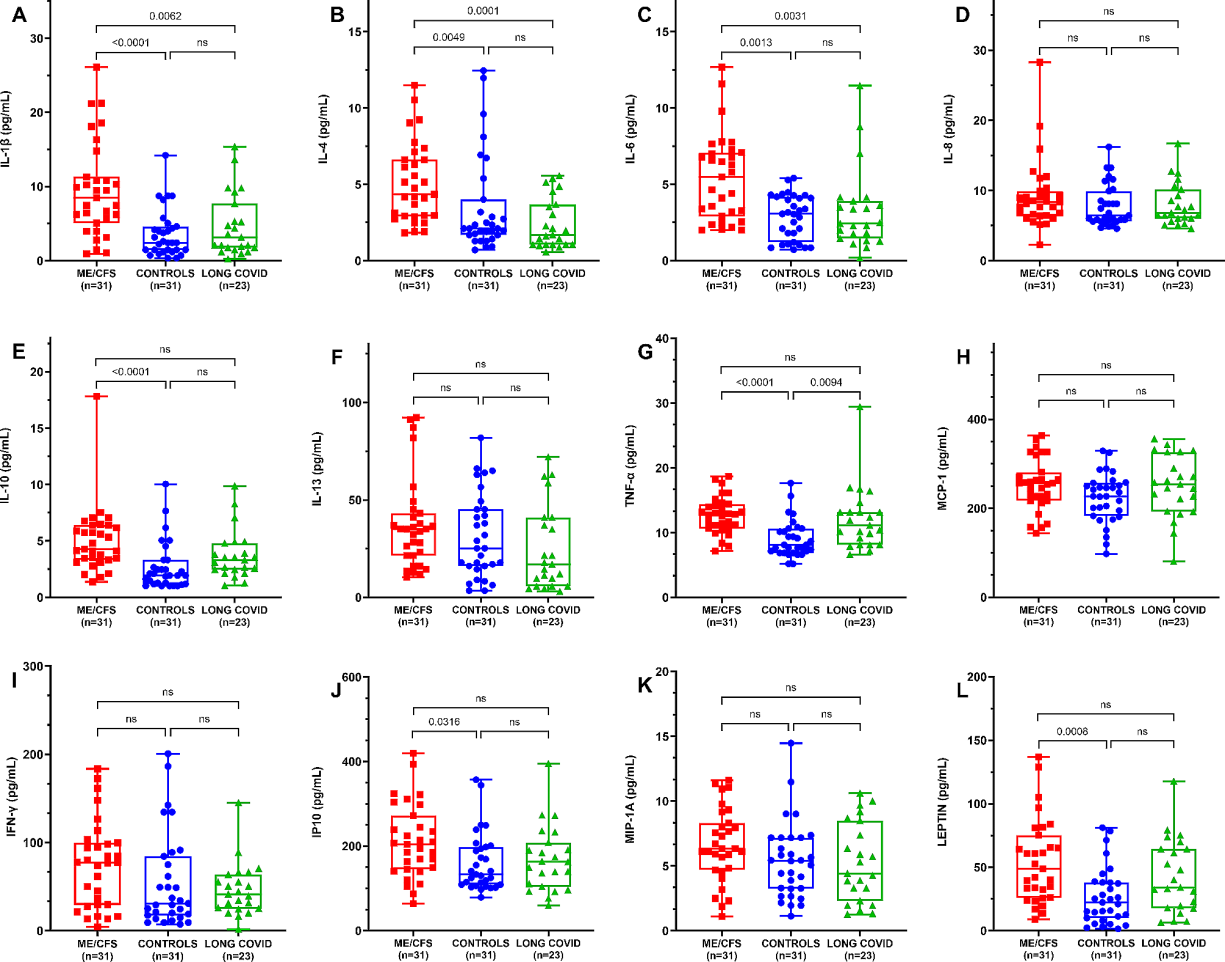
Measurement of circulating inflammatory cytokine/chemokine biomarkers in the study participants. Box plot analysis of twelve inflammatory cytokines/chemokines at baseline were assayed in serum from individuals with ME/CFS and long COVID and compared with matched sedentary healthy controls, as described in the *Methods* section (**panel A-L**). Each dot represents a single participant in each group. Values are shown as mean ± SEM of duplicates and are representative of two independent experiments. The box extends from the 25th to 75th percentiles, the line represents the mean, and the whiskers indicate the minimum and maximum values within SEM. Comparisons between cohorts were analyzed using the non-parametric Kruskal-Wallis test with Dunn’s post-hoc multiple comparison test. Significance level was set at *p < 0.05, **p < 0.01, and ***p < 0.001

Figure 3 shows mean values for plasma levels of the studied cytokines/chemokines related with inflammation. It can be seen that levels of TNF-α were higher in both the ME/CFS and long COVID groups, in comparison with matched sedentary healthy controls (both *p* < 0.01). Furthermore, compared with matched sedentary healthy controls, ME/CFS patients had higher levels of IL-1β (*p* < 0.005), IL-4 (*p* < 0.005), IL-6 (*p* < 0.005), IL-10 (*p* < 0.001), and IP-10 (*p* < 0.05). No differences among the three cohorts were found for IL-8, IL-13, MCP-1 or IFN-γ. No significant differences were shown in the inflammatory cytokines/chemokines profile between long COVID patients and matched sedentary healthy controls.

### Analysis of associations between clinical variables and circulating biomarkers revealed differences between ME/CFS and long COVID

#### Correlation analysis

**Fig. 4:**
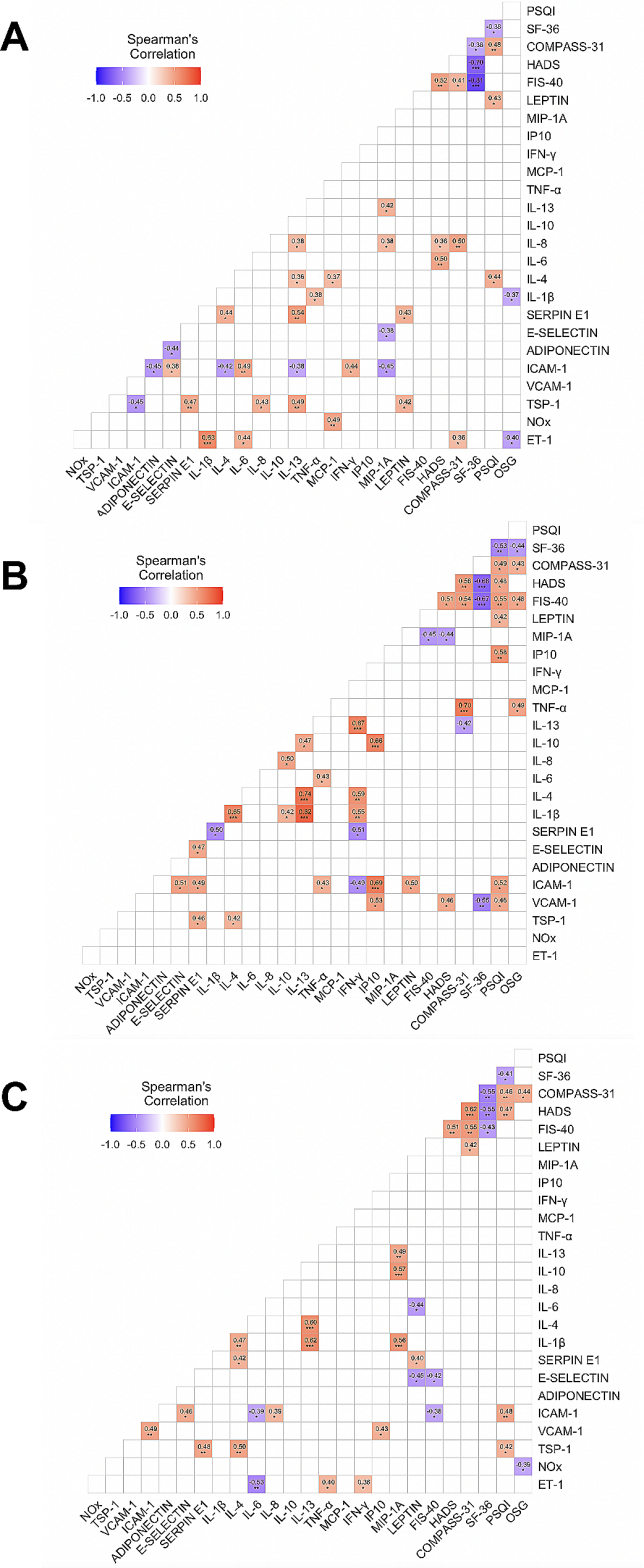
Correlation heatmap for study participants. Correlograms depicting Spearman’s correlation coefficients between biomarkers of endothelial function and inflammatory cytokines/chemokines status, and illness severity (assessed by self-reported outcome measures) of patients with ME/CFS (**A**), long COVID (**B**), and matched sedentary healthy controls (**C**) were illustrated using the Spearman’s rank correlation test and FDR-adjusted *p-value* < 0.05. Pairwise Spearman’s rank correlation coefficients (rho) are depicted for each correlation and represented by a color intensity scale (at the top left of each panel). Heat colors show standardized Z-scores (adjusted rho) across biomarkers and outcome measures. The color intensity is proportional to the strength of the association (rho-value), ranging from red (positive correlation) to blue (negative correlation). Significance was assessed using the Kruskal-Wallis test. FDR was calculated using the Benjamini-Hochberg method. Statistical significance level was set at *p < 0.05, **p < 0.01, and ***p < 0.001

Spearman’s correlation analysis was performed separately between data sets for the three groups of individuals (Fig. 4A-C). Study of the association between plasma cytokines/chemokines (inflammatory variables) revealed 11 positive associations in long COVID subjects, 6 in ME/CFS patients, and 6 in matched sedentary healthy controls.

Correlation analysis of endothelial biomarkers indicated that ICAM-1 was associated with VCAM-1 in controls, but not in either of the other two groups. Interestingly, all the significant associations between these variables were positive, except for levels of adiponectin in ME/CFS patients, which showed a negative association with E-selectin and ICAM-1.

Correlations between endothelial and inflammatory variables were more numerous in the ME/CFS group: total of 15 significant correlations in ME/CFS subjects, compared with 10 in matched sedentary healthy controls and 8 in the long COVID group. Of these, the most relevant were: (a) in ME/CFS patients, serpin E1 (PAI-1) was associated positively with IL-4 and IL-13; ICAM-1 was associated positively with IL-6 and IFN-γ and negatively with IL-4, IL-13, and MIP-1 A; ET-1 was associated with IL-1β and IL-6; (b) in the long COVID group, ICAM-1 was associated positively with IP-10 and TNFα and negatively with IFN-γ; serpin E1 (PAI-1) was associated negatively with INF-γ and IL-1β; (c) in matched sedentary healthy controls, ICAM-1 was associated positively with IL-8 and negatively with IL-6; ET-1 was associated positively with IFN-γ and TNF-α and negatively with IL-6.

Regarding clinical symptoms (assessed by outcome measures) and inflammation status variables (cytokines/chemokines profile), we found association between TNF-alpha and COMPASS-31 and OGS in long COVID. However, in the ME/CFS patients, four positive associations were found between symptomatology and IL-4, IL-6, and IL-8. Negative correlations were found for IL-1β and OGS in ME/CFS while IL-13 for COMPASS-31 in long COVID.

Regarding the association between clinical variables and endothelial variables, the most relevant is that clinical symptoms were associated with VCAM-1 in long COVID patients and with ET-1 in the ME/CFS group. In matched healthy controls, questionnaire scores were very low (or high in the case of the SF-36) and hence we do not consider these associations to be relevant.

### Principal component analysis

**Fig. 5:**
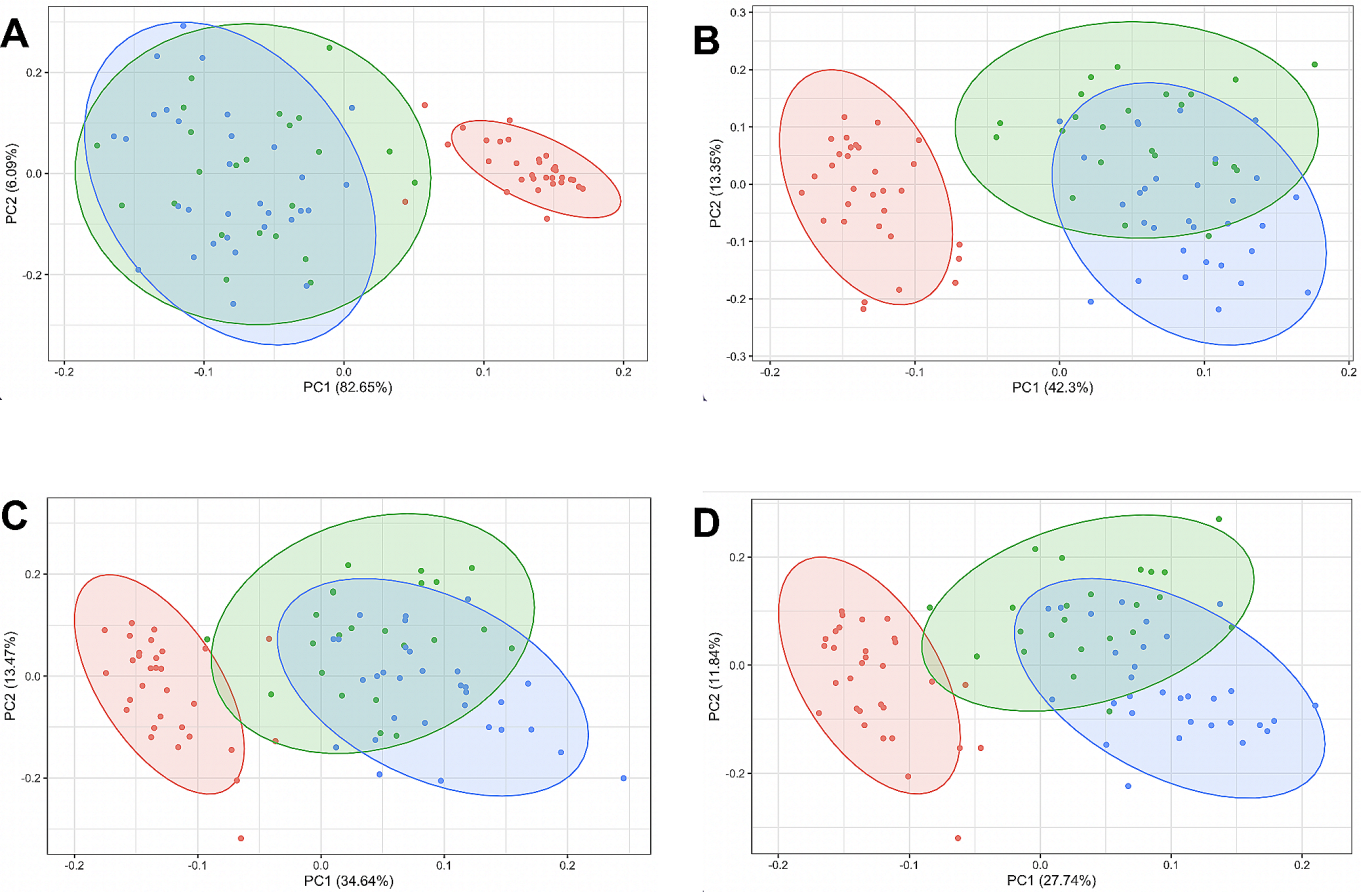
Principal component analysis between self-reported outcome measures and endothelial and inflammatory status biomarkers in the study participants. Principal component analysis (PCA) biplot of individuals and explanatory variables for each cohort of participants for the first (X-axis) and second (Y-axis) principal components. The biplot shows PCA scores with 95% confidence ellipses for each cohort of outcome measure scores (**A**), symptom questionnaire scores plus endothelial biomarkers (**B**), outcome measure scores plus inflammatory biomarkers (**C**), and outcome measure scores plus biomarkers of endothelial function and inflammatory status (**D**) in individuals with ME/CFS, long COVID, and matched sedentary healthy controls. Colored concentration ellipses (size determined by a probability level at 0.95) show the observations grouped by mark class

The principal component analysis (PCA) depicted in Fig. 5 is based on datasets from clinical and biochemical variables. The analysis of outcome measures revealed that the first two principal components explained 88.7% of the total variance within the dataset (PC1: 82.7% and PC2: 6.1%, as shown in Fig. 5A). This analysis yielded two significant clusters, effectively distinguishing the patient groups (ME/CFS and long COVID) from the healthy control group. At the group level, patients (with ME/CFS or long COVID) displayed a notably more scattered distribution in the PCA plot compared with matched healthy controls, confirming the greater data variance. However, there seemed to be no distinctive difference between the patient groups (Fig. 5A). Indeed, the PCA plot showed substantial overlap (around 80%) between the two patient groups, rendering them indistinguishable in terms of clinical questionnaires.

As an additional and independent approach, we explored whether a combination of endothelial and/or inflammatory biomarkers could aid in distinguishing between patients with ME/CFS and those with long COVID. To assess the impact of phenotypes, we conducted PCA to maximize the separation between the ME/CFS and long COVID groups, using the results of the clinical questionnaires as a starting point and introducing the studied biomarkers (Fig. 5B-D). The first two principal components explained 55.65% (PC1: 42.3% and PC2: 13.4%; Fig. 5B) 48.11% (PC1: 34.6% and PC2: 13.5%; Fig. 5C), and 39.58% (PC1: 27.7% and PC2: 11.8%; Fig. 5D) of the total variance within the datasets for endothelial, inflammatory, and combined endothelial and inflammatory biomarkers, respectively.

In the annotated plot, there was minimal overlapping between the control group and the patient clusters. Within the patient subset, we found that a subgroup of long COVID patients could be distinguished from ME/CFS patients (Fig. 5B, C and D). Specifically, the analysis suggested that incorporating endothelial biomarker data could correctly distinguish 51% of patients with ME/CFS from those with long COVID, while under the same conditions the inclusion of inflammatory biomarkers only classified 35% of patients (Fig. 5C). Finally, the incorporation of both endothelial and inflammatory biomarkers improved the classification results to 59% (Fig. 5D).

### Discriminant analysis

In an attempt to identify the optimal panel that could serve as markers to differentiate ME/CFS patients from the long COVID and healthy control groups, we performed a discriminant function analysis including only those biomarkers that varied significantly among study groups (ET-1, TSP-1, VCAM-1, serpin E1 (PAI-1), E-selectin, NOx, IL-1β, IL-4, IL-6, IL-10, and TNF-α), as well as scores on the patient-reported outcome measures (FIS-40, HADS, COMPASS-31, PSQI, OGS, and SF-36).

Discriminant analysis revealed two canonical discriminant functions: the first explained 88.9% of the variance, canonical R^2^ = 0.91, whereas the second only explained 11.2%, canonical R^2^ = 0.57. In combination these discriminant functions significantly differentiate the groups (Wilks’ lambda = 0.38; χ^2^ = 239; *p* < 0.001). Overall, 95.3% of the original grouped cases (87.1% of ME/CFS patients, 100% of those with long COVID, and 100% of matched sedentary healthy controls) were correctly classified. If the same procedure was carried out with stepwise variable selection, the final model included serpin-E1 (PAI-1), NOx, IL-1β, IL-6, and FIS-40 (Wilks’ lambda = 0.068; χ^2^ = 215; *p* < 0.001) and 85.9% of participants were correctly classified (67.7% of ME/CFS patients, 91.3% of those with long COVID, and 100% of matched sedentary healthy controls).

## Discussion

Long COVID exhibits significant overlap with ME/CFS in immunological, neurological, and mitochondrial dysfunction [16, 34], and the two have certain similarities in their pathophysiological mechanism, including immune dysregulation, a hyper-inflammatory state, oxidative stress, and autoimmunity [35]. This overlap raises the question of whether long COVID predisposes individuals to ME/CFS or if the two represent distinct pathological conditions.

To our knowledge, this is the first study to evaluate outcome measures and circulating biomarkers of endothelial function and inflammation in ME/CFS and long COVID from Spain. Here we investigated candidate biomarkers for distinguishing between the two subgroups within ME/CFS, particularly markers associated with endotheliopathy and low-grade systemic inflammation. Our study is significant as it provides objective biological data for differentiating ME/CFS patients from both individuals with long COVID and healthy sedentary controls.

Notably, and in comparison with matched sedentary healthy controls, the collective cohort of patients (long COVID and ME/CFS) exhibited elevated levels of ET-1, VCAM-1, and TNF-α, as well as reduced levels of NOx, signifying underlying inflammation and endothelial dysfunction. However, long COVID patients differed from those with ME/CFS in having lower levels of TSP-1, serpin E1 (PAI-1), E-selectin, IL1-β, IL-4, and IL-6. It should be noted that some of these molecules, such as VCAM-1 and E-selectin, are adhesion molecules involved in the interplay between endothelial dysfunction and inflammation [36, 37]. VCAM-1 expression occurs in both large and small blood vessels post-stimulation of endothelial cells by cytokines, and notably in response to TNF-α [38]. This suggests a potential association between ME/CFS and heightened ET-1 mediated vasoconstriction, indicated by diminished nitrogen oxide levels [11]. A study conducted by Prof. Warlé [39] revealed correlations between ET-1 levels and long COVID symptoms at 2 years after acute COVID-19 infection. Under physiological conditions, ET-1 production is small and bioavailability of nitric oxide (NOx) is preserved, inducing vasorelaxation. However, increased ET-1 levels can play a pathogenic role in vascular dysfunction and the subsequent development of cardiovascular disorders by NOx modulation in patients with ME/CFS and long COVID. As a result, selective and dual ET receptor antagonists could provide therapeutic benefits because they may induce increased NO bioavailability and mitigate redox imbalance which could in turn improve endothelial function in these conditions.

With specific regard to the ME/CFS cohort, our results revealed higher levels of both pro-inflammatory (IL1-β, IL-6, TNF-α, and IP-10) and anti-inflammatory cytokines (IL-4, IL-10), as compared with matched sedentary healthy controls, indicating an imbalanced cytokine profile and disturbed immune system. IL1-β is a pro-inflammatory cytokine and plays a role in the immune response to infections and injury, while TNF-α, also pro-inflammatory, is involved in inflammation and cell death. Cytokines are essential small proteins regulating cellular signaling, inflammation, and immune responses, and thus they potentially influence chronic pain [40]. The predominance of pro-inflammatory molecules observed in ME/CFS patients may directly influence the synthesis and secretion of serpin E1 (PAI-1), levels of which were also higher in ME/CFS than in controls, leading to increased levels of circulating serpin E1 (PAI-1) [41]. An increased serpin E1 (PAI-1) level is a common determinant during infection that is frequently associated with a hypofibrinolytic state and thrombotic complications, as well as being a common feature of metabolic syndrome in chronic conditions [41].

Studies have shown that cytokines are released during the cytokine storm following acute COVID-19 infection, as well as during its post-acute sequelae [42, 43]. However, it is intriguing that in our long COVID cohort, only the cytokine TNF-α showed higher levels than in matched sedentary healthy controls. Furthermore, the long COVID group differed from ME/CFS patients in having lower levels of TSP-1, serpin E1 (PAI-1), E-selectin, IL-1β, IL-4, and IL-6, suggesting a lower level of inflammation within the former cohort. This may be due to shorter illness duration (2 years) in the long COVID cohort at the time of the study.

Laboratory findings and circulating biomarkers in long COVID have been extensively reviewed, but without a complete consensus being reached. Some studies highlight ET-1, IL-6, TNF-α, and MCP-1 as pivotal biomarkers for classifying clinical manifestations [15]. Others, however, suggest that a year after discharge, patients who had contracted COVID-19 did not exhibit significant differences in serum IL-6, VCAM-1, ICAM-1 or P-selectin in comparison with matched sedentary healthy controls [44]. These findings, together with our results, suggest that the cytokine storm commonly observed in post-COVID infection may not persist beyond a two-year period, although endothelial dysfunction does seem to be present. In this regard, the correlation between cytokines and endothelial function markers provides valuable insight into the networks of interactions among signaling molecules. Our analysis showed that the association between endothelial biomarkers and cytokines or inflammatory variables was stronger in the ME/CFS cohort, compared with long COVID patients or matched sedentary healthy controls.

The vascular endothelium, the innermost layer of blood vessels, plays a pivotal role in regulating vascular tone, cellular adhesion control, smooth muscle cell proliferation, and macromolecule transport across vessel walls. Endothelial dysfunction arises from an imbalance between vasodilators and vasoconstrictors produced by endothelial cells, leading to vasoconstriction, leukocyte trafficking, inflammation, and coagulation-thrombosis [45]. Elevated levels of E-selectin, ICAM-1, VCAM-1, and serpin E1 (PAI-1) are indicative of endothelial inflammation and injury [46].

Endothelial activation has been reported to be triggered by several stimuli, including bacterial endotoxins and inflammatory cytokines such as TNF-α, ILs, and IFN-γ, and it has been identified as a common feature in both ME/CFS and post-COVID-19 condition [11, 36]. In the context of COVID-19, endothelial dysfunction has been implicated in its pathogenesis [46–48]. Acute COVID-19 infection disrupts vascular homeostasis by directly infecting endothelial cells via ACE2 receptors, while inflammatory mediators contribute to endothelial injury [45]. Post-infection, excessive production of inflammatory mediators such as IL-1β, IL-6, IL-8, TNF-α, MCP-1, and IP-10, as well as the presence of endothelial inflammation markers (IL-6, TNF-α, ICAM-1) in lung tissues, have also been reported [49, 50]. This chronic endothelial dysfunction could potentially contribute to the observed higher BP levels in ME/CFS patients, as reported previously [22]. In our study, however, it remains uncertain whether a similar pattern exists in long COVID patients. The mean time lapse for our long COVID patients following infection was approximately two years, raising the possibility that the described pattern may have dissipated, with cytokine levels potentially returning to normal. Moreover, in the context of ME/CFS, where endothelial dysfunctions may emerge as the disease progresses, it is plausible that immune and inflammatory responses are chronically altered, whereas in long COVID patients, with fewer years of disease, these responses might still be evolving [51].

The relationship between circulating biomarkers and symptomatology in ME/CFS and long COVID remains incompletely understood. In the present study, we found that symptomatology in general was differentially associated with biomarkers according to the patient group. For instance, in the case of ME/CFS, symptomatology (assessed by the HADS, COMPASS-31, and PSQI) was positively associated with inflammatory markers such as ET-1, IL-4, IL-6, IL-8, and leptin, whereas this was not the case for long COVID patients. Interestingly, despite there being no significant differences in plasma MCP-1 levels between the groups, this variable was correlated with clinical symptoms in the long COVID cohort. Although symptomatology cannot be attributed to a particular molecule, there is a general association in patients between cytokine imbalance and symptomatology that is not observed in matched sedentary healthy controls. Thus, while it has been suggested that cytokine levels have limited potential as biomarkers for ME/CFS [52], determining these levels remains crucial as it provides evidence of immune system alterations and insights into patients’ inflammatory status. Our findings demonstrate that plasma cytokine/chemokine levels not only differentiate between matched sedentary healthy controls and patients but also between the ME/CFS and long COVID cohorts, suggesting distinct immune responses across the groups.

A notable finding in the long COVID cohort was the significantly lower levels of TSP-1. This molecule, which is involved in mediating endothelial cell apoptosis and inhibiting angiogenesis, is secreted by activated platelets, and platelet activation can be influenced by interleukins [37, 53]. In the context of long COVID, it is plausible that endothelial inflammation, even in the absence of significantly elevated cytokine levels, might have affected platelet function, leading to reduced TSP-1 secretion. Therefore, it is conceivable that although COVID-19 patients may have significantly higher TSP-1 levels than matched sedentary healthy controls two years after the acute following the infection, the lower levels of TSP-1 they exhibited could be due to impaired platelet activation [54].

The results of the PCA strongly indicate the potential for differentiating between the three groups using a combination of endothelial, inflammatory, and clinical variables. Specifically, the findings suggest that while symptomatology may appear similar in the two patient groups, outcomes from clinical questionnaires effectively distinguished patients from matched sedentary healthy controls, although they fell short of distinguishing between the long COVID and ME/CFS groups. Our study nevertheless underscores the significance of inflammation and endothelial dysfunction-related biomarkers in distinguishing patients with ME/CFS following SARS-CoV-2 infection from other patient groups.

Importantly, discriminant analysis involving just four molecules (serpin E1/PAI-1, NOx, IL-1β, and IL-6) and FIS-40 scores was able to correctly classify a striking 85.9% of participants. Specifically, the analysis showed that clinical symptomatology and NOx levels are key to distinguishing between patients and matched sedentary healthy controls, whereas the variance between ME/CFS and long COVID is explained largely by the two pro-inflammatory molecules, IL-6 and IL-1β, along with serpin E1 (PAI-1), considered an endothelial biomarker associated with senescence. A refined subset of four markers and clinical FIS-40 scores therefore enable significant differentiation between these groups. Obviously, these findings await validation in a larger dataset to ascertain their potential clinical utility for diagnostic or treatment purposes.

To sum up, this study evidences the correlation between inflammation markers and endothelial biomarkers in ME/CFS, including a subset of patients with long COVID, and confirms the presence of inflammation and endothelial dysfunction in both conditions (ME/CFS and long COVID). The results also show that the inflammatory response, reflected in cytokine levels, is stronger in ME/CFS and likely contributes significantly to the development of endothelial dysfunction. However, it is essential to recognize that endothelial dysfunction is a multifaceted process, and normal cytokine levels do not rule out other factors contributing to dysfunction. Accordingly, long COVID and ME/CFS might have differing origins of endothelial dysfunction. Although long COVID shares endothelial dysfunction with ME/CFS, it lacks the sustained high cytokine levels observed in ME/CFS. Therefore, enhancing endothelial function may be an additional step in mitigating the associated morbidities observed in ME/CFS.

### Limitations of the study

The current study has some limitations. The small sample size and the fact that both ME/CFS and long COVID are multifactorial conditions characterized by their great heterogeneity and phenotypic complexity among participants may possibly have reduced the statistical power and the chance of detecting true consequences. This might especially concern the analyzes concerning the impact of the preliminary panel of putative circulating diagnostic biomarkers to distinguish ME/CFS from long COVID. Future studies are required to confirm the association between outcomes measures and circulating putative biomarkers in larger populations to increase the confidence and the statistical power of the analysis.

Furthermore, the limited diversity of potential covariates (confounders) in the available data reduced the number of possible factors of interest to adjust for in the correlation analyzes. In addition, the inability to report separate sex-specific association due to a low number of males in both the long COVID and ME/CFS groups. This is relevant because the disease burden of ME/CFS is higher in females, and recent studies have uncovered sex differences in its pathophysiology. The inclusion of both sexes in our sample nevertheless provides a comprehensive picture of disease characteristics, which may be regarded as a strength of the study. A further limitation is that we only report correlations, underscoring the need for further research to establish causal relationships between the variables considered. Similarly, our use of an observational cross-sectional design and the differing duration of illness in the two patient groups (7 years for ME/CFS and 2 years for long COVID) mean that more comprehensive longitudinal research is required to achieve a better understanding of disease progression in the two scenarios. Indeed, further research is crucial for unraveling the pathomechanism of endothelial dysfunction and inflammation and their role in classifying these two distinct conditions.

Another limitation of the study is that illness severity were assessed through self-report questionnaires and reflect the week prior to follow-up assessments. Moreover, the use of self-reported outcome measures to assess the natural course of illness (symptoms) is prone to recall bias and over-reporting. Furthermore, we measured outcomes that were reported in structured medical coding in electronic health records and we had no access to diagnoses and outcomes reported in free text format. These data may not, therefore, completely reflect diagnoses and reported outcomes from study population. Although we cannot rule out diagnostic errors relating to conditions with similar health outcomes, we believe that these are equally likely in both groups, in other words, misclassification of outcomes is non-differential. Patient-reported outcomes such as weakness, cognitive impairment, anosmia, and disgeusia are also less objective than are clinical diagnoses by physicians and might not be uniform and accurate.

Importantly, we cannot rule out potential behavioral and environmental factor differences between infected and uninfected people, which might cause overestimation of incidence among the infected population. Neither can we exclude the possibility of additional confounders affecting long-term outcomes of acute SARS-CoV-2 infection that were unavailable to us in this study. Furthermore, some outcomes were reported at low frequency and larger populations might be necessary to gain a more reliable picture.

## Conclusions and future perspectives

This study investigated the correlation between circulating putative biomarkers of endothelial dysfunction and inflammation status in ME/CFS and long COVID. The findings confirm the presence of low grade systemic inflammation and endothelial dysfunction in both conditions. Patients with ME/CFS display a heightened inflammatory response profile, particularly in plasma cytokine/chemokine levels, and this likely plays a significant role in the development of endothelial dysfunction. While pro-inflammatory cytokines such as IL-6 and TNF-α directly impact endothelial function, they may not be the sole determinants, and other additional factors may also contribute to impaired endothelial function in this condition.

Our results also suggest that ME/CFS and long COVID may have different origins of endothelial dysfunction, insofar as long COVID patients lack the sustained high cytokine/chemokine levels that are observed in ME/CFS. Enhancing endothelial function may therefore be an additional step in addressing the associated health issues in ME/CFS. Our data may provide a rationale for the selection of novel therapeutic strategies for further interventions. Further research is needed to understand the specific pathomechanisms underlying autonomic dysfunction and low grade systemic inflammation in individuals with ME/CFS and long COVID.

## Data Availability

All data supporting the findings of this study are available within the paper. However, the individual data of patients participating to this study are not openly available due to reasons of sensitivity (GDPR under European requirements) and are available from the corresponding author upon reasonable request. The datasets used and/or analyzed during the current study are available from the corresponding author upon reasonable request.

## Abbreviations

BMI: Body mass index
COPD: Chronic obstructive pulmonary disease
CKD: Chronic kidney disease
DBP: Diastolic blood pressure
ET-1: Endothelin-1
E-selectin: Endothelial selectin
HR: Heart rate
IBS: Irritable bowel syndrome
ICAM-1: Intercellular adhesion molecule-1
IFN−γ: Interferon gamma
IP-10: Interferon gamma-induced protein-10
MCP-1: Monocyte chemoattractant protein-1
ME/CFS: Myalgic encephalomyelitis/chronic fatigue syndrome
MIP-1 A: Macrophage inflammatory protein-1 A
NOx: Nitrite/nitrate levels
NLT: 10-min NASA lean test
NPP: Narrowed pulse pressure
OGS: Orthostatic grading scale
OI: Orthostatic intolerance
OH: Orthostatic hypotension
POTS: Postural orthostatic tachycardia syndrome
PP: Pulse pressure
PASC: Post-acute sequelae of COVID-19
PAI-1 (Serpin E1): Plasminogen activator inhibitor-1
SBP: Systolic blood pressure
TNF-α: Tumor necrosis factor alpha
TSP-1: Thrombospondin-1
VCAM-1: Vascular cell adhesion molecule-1
SARS-CoV-2: Severe acute respiratory syndrome coronavirus 2
